# Attachment and patient activation as predictors of the interest and use of telemedical health applications –results of an observational study in primary health care

**DOI:** 10.1186/s12875-022-01711-0

**Published:** 2022-04-29

**Authors:** Katja Brenk-Franz, Leo Johannes Leonhardt, Bernhard Strauß

**Affiliations:** Institute of Psychosocial Medicine, Psychotherapy and Psychooncology Jena University Hospital, Friedrich-Schiller-University Jena, Stoystrasse3, 07740 Jena, Germany

**Keywords:** Adult attachment, Patient activation, E-health care, Primary care, Telemedicine

## Abstract

**Background:**

Telemedicine applications are becoming more accepted and offer great potential to support physicians and patients. However, there is an increasing need for research, especially in personal predictors that determine the interest and use of telemedicine and e-health applications. This study aims to identify if attachment and patient activation are potential predictors of the interest in and the use of e-health applications in primary care patients.

**Methods:**

We used data from the cross-sectional observational Weimar TelMed study on 192 patients treated by general practitioners from a practice of family medicine in Thuringia, the middle of Germany. The adult attachment was measured using the ECR-RD12 and patient activation with the PAM-13D. Multiple regression analysis by the General Linear Model was used to evaluate the association between attachment, patient activation, and interest in and use of e-health applications.

**Results:**

Patient activation was associated with a higher interest in e-health care. The attachment dimension avoidance was a potential predictor of interest in e-health and e-health-care use.

**Conclusion:**

Adult attachment is an essential predictor of different ways of healthcare use. While avoidant patients evade contact with general practitioners, self-determined access via e-health seems to improve the health care of these patients. A personalized view might be a basis for the evaluation of individual approaches in Primary Care.

## Background

The rapid and global development of information and communication technology with the trend towards an increasing development of powerful mobile devices, as well as the associated powerful application software (apps), is also influencing the health sector. So-called health applications, in particular, are enjoying increasing attention. However, there is an increasing need for research into personal factors that can inhibit or promote the acceptance and use of such e-health. According to the WHO definition, e-health (electronic health) describes the use of information and communication technologies to promote general health and health-related areas [1].

E-health solutions and apps for therapy monitoring or as a diagnostic tool or even video consultation are encountering growing interest in the population. The current results of a population-representative study in Germany showed that 33% of patients have used the opportunity to make an appointment with a doctor online. 26% have an app on the subjects of nutrition, exercise and relaxation or have taken part in an online course [2].

Since health-related apps can support health care but are not of interest to all patient groups, factors should be identified that determine the interest and use of such mobile health-related applications. Access to, and appropriate use of, health-related information is essential for coping with illness and maintaining good health. The knowledge gained in this way serves as the basis for health-related decisions and helps patients cope with emotional stress and subjectively perceived insecurity [3]. Explanatory approaches for health-related information action offer the first indications of relevant predictors [4–6]. So far, however, there are only relatively few models that explain health-related e-health use and no uniform understanding of the relevant predictors. Accordingly, special interest should be placed on the question of whether specific patient characteristics (e.g. attachment and patient activation) lead to an interest in and use of telemedical health applications among primary care patients.

The factors influencing searches for health-related information on the Internet and the use of e-health applications so far identified as relevant comprise personal characteristics such as age, gender and level of education [7–10]. In addition, the current state of health is a situational trigger for information needs and initial studies indicate that the Internet is primarily a source for people who are particularly interested in health issues (highly involved or activated) and patients with chronic conditions [11]. Ernsting et al. reported that health app users were younger, performed more searches on the Internet and had a higher level of health literacy than non-users. The results are from a population-based survey (*N* = 4144) among Germans [12]. There are conflicting results regarding the assumption of a more physically active and healthier lifestyle by health app users [13–15].

Both the search for health-related information and the use of health-related apps are continuously increasing. Current acute or chronic illnesses of the patients are important motivational factors influencing online research for symptoms and treatments [7, 16].

### Adult attachment

Attachment theory provides a model to describe individual differences in interpersonal proximity-distance regulation and affect regulation. These are based on early childhood experiences with primary caregivers. The patterns are activated in situations perceived as threatening, such as acute or chronic disease [17, 18]. Attachment theory explains differences between individuals in terms of the activation of their attachment system. The present research refers to the dimensional measurement of attachment using the dimensions anxiety and avoidance [19, 20]. Avoidance patients feel uncomfortable about talking about their feelings. Avoidance patients are more likely to suppress the basic needs for closeness and intimacy. They often avoid personal GP contacts to dodge threatening health information [21]. Anxious patients have an overactivated attachment system that increases their feelings. They are afraid of abandonment, rejection or being alone. They are often emotionally preoccupied in relationships [22]. We hypothesized that patients with avoidant attachment through their increased striving for autonomy will report more interest in and use of e-health applications.

### Patient activation

The measurement of patient activation is becoming increasingly relevant in the assessment of patient-relevant outcomes. It has conceptual overlaps with empowerment and self-management [23]. For primary care, it is becoming increasingly important to have an active, informed patient as a partner in health management and health-related behaviour change for example also in using e-health applications [24]. Patient activation is now considered a good predictor of changes in health-related behaviours and to continue these behaviours independently [25, 26].. Specifically, chronically ill patients with high patient activation have better self-management skills and behaviour. They adequately implement health-related behaviours such as physical activity and health-related diet. Active patients reported higher scores in quality of life, health-related outcomes, self care, and lower scores in use of health care [27, 28]. Based on these predictors, i.e. attachment characteristics and patient activation, the aim of the article is to contribute to a better understanding of the interest and use of digital health offers in the form of information searches on the Internet and the use of health apps.

**Fig. 1:**
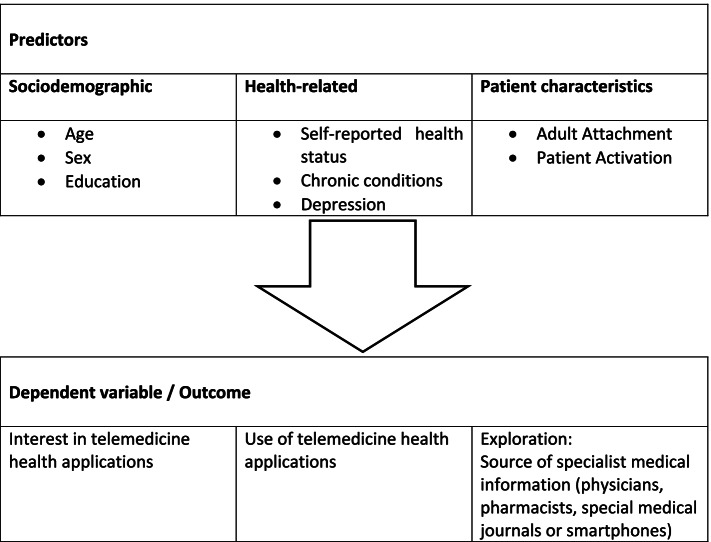
Overview of the predictors and outcome variables to be examined

The primary objective is to investigate the extent to which sociodemographic characteristics (age, gender, education), disease-specific characteristics (self-reported health status, number of chronic conditions, patients’ psychological characteristics), and patient characteristics (retention and patient activation) influence interest in and use of telemedicine health applications among patients receiving primary care (see Fig. 1). Secondary, it should be exploratively examined which influence these predictors have on the relevance of different medical information sources (physicians, pharmacists, journals or the Internet).

## Methods

### Data collection

Data collection was performed by self-rating questionnaires with tablets in primary care setting in the Weimar TelMed-Study in 2017, a cross-sectional observational study. Four of the physicians of the practice were all specialists in general medicine or internal medicine. One of the physicians was GP assistant doctor. The practice is a medical practice of family medicine in the middle of Germany (Thuringia) All patients were approached in the practice on fixed days. The eight fixed survey days were mainly Tuesday and Thursday because there are long office hours in the practice. Written informed consent was obtained from all the participants. To be eligible, a subject had to be at least 18 years old and be a patient at the primary care practice. We excluded emergency patients and patients with blindness, deafness, or insufficient German language skills or without informed consent. Health care assistants informed all eligible patients about the study and asked them if they would participate in the study. They then handed out the tablet with the questionnaires in opaque envelopes to ensure blinded data entry. We provided no financial incentive.

### Ethics approval and consent to participate

The study was conducted in accordance with the ‘Declaration of Helsinki’, the guidelines of Good Clinical Practice and was approved by the institutional review board of the University Hospital Jena (No.5002/121616). All the participants have given written consent to participate.

### Predictor variables

#### Adult attachment

Adult attachment was assessed using the Experiences in Close Relationships (ECR-RD12), a 12-item scale that has been validated against the longer ECR-RD-36 scale [19, 22]. This measure yields anxious (ECR-RD12anx) and avoidant (ECR-RD12avoid) relationship style scores based upon the scoring of six items related to each style. The questionnaire uses a seven-point Likert scale. Higher scores indicate higher attachment insecurity in these respective areas. Higher scores in avoidance tend to represent avoidance of closeness and less openness. Attachment anxiety tends to represent fear of abandonment or rejection. The Cronbach’s alpha reliability scores are .91 and .92 for the two subscales [29].

#### Patient activation

Patient activation was measured with the PAM13-D. The PAM13-D is a valid short version of the PAM [30] and consists of 13 items on a four-point Likert scale [31, 32]. Each item has four response categories with scores from 1 (strongly disagree) to 4 (strongly agree). Patient activation is measured by a sum score ranging from 13 to 52. Higher scores represent higher levels of patient activation. Then a standardised transformation into a score from 0 to 100 is performed. The PAM13-D has been shown to have good construct validity and internal consistency (Cronbach’s alpha = .86 in primary care setting) [32].

### Demographic variables

Patients were asked about their age, sex, family status and education level. The questionnaire was based on the references of the Association of Epidemiology [33].

### Health condition

The primary care patients completed a list of predefined chronic conditions based on the list of chronic diseases of the MultiCare cohort study [34]. In addition, we used the EuroQol VAS to measure the patient’s self-rated health on a 20-cm- vertical, visual analogue scale with two endpoints on a 100-point scale labelled ‘the best health you can imagine’ and ‘the worst health you can imagine’ [35].

### Depression

The PHQ-9 is the 9-item depression module from the Patient Health Questionnaire, and a widely used instrument for measuring the severity or levels of depression in patients in clinical practice and primary care. This self-reported scale measuring the symptoms of major depression is derived from the Diagnostic and Statistical Manual (DSM-IV). The items can be scored from 0 (not at all) to 3 (nearly every day) and the sum score can range from 0 to 27. The PHQ-9 shows good validity in primary care settings [36].

### Interest and use of E-health application

Questions about telemedicine applications had to be developed, checked and modified before by a group of experts. The group consisted of two doctors and a psychologist. Studies and literature reviews were included. Items were created and checked for relevance and comprehensibility in a pre-test. With the scale *interest in e-health*, we measured directly the interest in the use of e-health-related applications (telemedicine, health applications), and we asked directly for the use of *health* (use of health-related Internet recherche and use of app-based applications). Interest in e-health means being interested in telemedicine and health care applications, being enthusiastic about them and being able to imagine using them in the future. Meanwhile, the use of e-health means actually working with e-health applications for example on the smartphone already at present. Higher scores on the scale indicate higher interest and user behavior. When asked about the importance of physicians, pharmacists, special medical journals or smartphones as a source of specialist medical information, a 4-point Likert scale was used to evaluate the results from “not at all” to “exclusively”.

### Statistical analysis

#### Data analysis

The data were analyzed using the Statistical Package for Social Sciences Statistics version (IBM, SPSS 23.0). Multivariate linear regression models (GLM) were used to identify whether attachment insecurity and patient activation were predictors of interest in e-health and use of e-health. Age, gender, education, and health conditions (VAS of self-rated health, number of chronic diseases, depression) were included in the models as covariates. Statistical significance was determined with *p* < 0.05.

## Results

### Sample description

One hundred ninety-two patients aged from 18 to 80 years were examined in the study (Table 1). Patients were diagnosed with between 0 and 23 chronic diseases. The most common chronic diseases within the study population were hypertension (44%), dorsopathies (32%), arthrosis (28%), spinal osteochondrosis (27%), thyroid disorders (24%), cardiac arrhythmias (17%) and disorders of lipoprotein metabolism (17%).

**Table 1:** Characteristics of the study sample (*N* = 192)

Variables	Categories		n	%
Age M = 46.3 SD = 16.4	< 20		13	6.8
	20–29		26	13.5
	30–39		29	15.1
	40–49		27	14.1
	50–59		53	27.6
	60 and older		44	22.9
Sex	Female		109	65.8
	Male		83	43.2
Marital status	married		88	45.8
	single		73	38.0
	divorced		19	9.9
	widowed		12	6.3
Education	Low		30	15.6
	Middle		98	51.0
	High		61	31.8
	missing		3	1.6
	**Min**	**Max**	**M**	**SD**
Number of chronic diseases	0	23	3.2	3.0
Health VAS	10	100	64.2	20.6
Depression PHQ-9	0	25	5.1	4.6
Patient activation PAM-D	13	52	42.7	7.0
Attachment Avoidance	1	7	2.4	1.5
Attachment Anxiety	1	7	2.4	1.4
Interest in e-health	1	4	2.4	1.9
Use of health	1	4	2.3	1.8
Source of special medical information				
Physicians	1	4	2.9	0.6
Pharmacists	1	4	2.0	0.8
Special medical journals for patients	1	4	1.9	0.8
Smartphones	1	4	1.9	0.8

*M* mean, *SD* Standard deviation, *%* Percentage, *VAS* Visual analogue scale, *PHQ* Patient Health Questionnaire, *PAM* Patient Activation Measure

The sociodemographic distribution characteristics of age, gender, marital status, education, number of chronic diseases, health status (VAS), depression (PHQ9), patient activation (PAM-D), attachment of the 192 patients in total who participated in the study are quite typical for a primary care sample [37–39]. It turns out that around two thirds of the respondents are over 40 years old (64.6%). We are dealing with a typical age distribution in a GP practice in the city area. The distribution of gender and education is similar to comparable studies in primary care [37, 38]. The number of excluded patients was small. There were no emergency patients on the days of inclusion. The proportion of patients with poor German language skills in the practice is around 1 %. This is below the German average and far below the proportion in metropolitan areas.

### Interest and use of eHealth applications

Multivariate regression analysis to examine the factors influencing the interest in telemedical health-related applications and the use of these telemedical health-related applications were calculated using the general linear model (GLM). In view of the risk of bias, the hypotheses were not calculated individually with ANOVAs but in multiple regression analysis models, so that the influence of the other variables could be adjusted in each case. Age, gender, education, state of health, number of chronic illnesses, depression, attachment and patient activation were examined as predictors. The results of the parameter estimates for the model including the regression coefficients and confidence intervals can be found in Tables 2 and 3.

**Table 2:** Predictors of interest in e-health related health applications

Predictors	B	SE	T	p	95%-CI	
**Age**	−.008	.006	−1.317	.190	−.020	.004
**Health VAS**	−.003	.004	−.821	.413	−.012	.005
**Number of chronic diseases**	.025	.029	.849	.397	−.033	.083
**PAM_D**	.038	.013	2.957	**.004**	.013	.063
**PHQ-9**	.014	.022	.639	.523	−.030	.059
**ECR-RD-12 Anxiety**	−.064	.062	−1.034	.302	−.186	.058
**ECR-RD12 Avoidance**	.468	.057	8.220	**.000**	.356	.581
**SEX, Female**	.111	.272	.408	.683	−.426	.648
**SEX, Male = 1**	0	.	.	.	.	.
**Education, Low**	.264	.356	.740	.460	−.440	.967
**Education, Middle**	−.043	.259	−.167	.868	−.554	.468
**Education, High**	0	.	.	.	.	.

*B* Beta coefficient, *SE* Standard error, *T t* test statistic, *p* Statistical significance, *CI* 95% confidence intervals, *VAS* Visual analogue scale, *PHQ* Patient Health Questionnaire, *PAM* Patient Activation Measure, *ECR-R* Experiences in Close Relationships-Revised

**Table 3:** Predictors of the use of e-health-related health applications

Predictor	B	SE	T	p	95%-CI	
**Age**	−.009	.004	−2.472	**.014**	−.016	−.002
**Health VAS**	−.001	.002	−.527	.599	−.006	.004
**Number of chronic diseases**	−.026	.017	−1.520	.130	−.059	.008
**PAM_D**	.003	.007	.425	.671	−.011	.017
**PHQ-9**	−.008	.013	−.594	.554	−.033	.018
**ECR-RD-12 Anxiety**	−.058	.035	−1.638	.103	−.128	.012
**ECR-RD12 Avoidance**	.203	.033	6.226	**.000**	.139	.267
**Sex, Female**	.290	.156	1.867	.064	−.016	.597
**Sex, Male**	0	.	.	.	.	.
**Education, Low**	.301	.204	1.474	.142	−.102	.703
**Education,**	.099	.148	.668	.505	−.193	.391
**Education, High**	0	.	.	.	.	.

*B* Beta coefficient, *SE* Standard error, *T t-test statistic*, *p* Statistical significance, *CI* 95% confidence intervals, *VAS* Visual analogue scale, *PHQ* Patient Health Questionnaire, *PAM* Patient Activation Measure, *ECR* Experiences in Close Relationships-Revised

Interest in telemedical health applications: A significant influence was found in patient activation and avoidant attachment. Patients with higher patient activation and levels of avoidant attachment were more interested in telemedical health applications. There was no influence of age, education or gender or influences of health factors (see Table 2).

Use of telemedical health applications: There was an influence of age and an influence of patient characteristics (see Table 3). Younger patients and patients with a higher degree of avoidant attachment reported more e-health usage behavior.

### Source of health information

The exploratory analysis using the GLM concerning the influence of a) socio-demographic characteristics (age, gender, education) b) disease-specific characteristics (number of chronic diseases, self-reported health status) and c) patient characteristics (attachment, patient activation and depression) on various sources for health-related information (physicians, pharmacist, magazines, books, internet) resulted in the following:

- No significant predictors related to the use of the physician as a source of specialist medical information were identified.
- However, there was a gender influence when the pharmacist was used as a source for medical information according to which this source is more relevant for women (B = 0.40, SE = 0.19, *p* < 0.05).
- Furthermore, there was a significant positive influence of age using medical journals for patients as a source of medical information (B = 0.016, SE = 0.005, *p* < 0.001).
- When using a smartphone in relation to health issues, there was a negative age effect (− 0.038, SE = 0.006, *p* < 0.001) and a positive effect concerning patient activation (B = 0.051, SE = 0.02, p < 0.001) and attachment avoidance (B = 0.26, SE = 0.05, p < 0.001) of patient loyalty. Education or gender had no significant influence.

## Discussion

With regard to the hypotheses, significant influences of the predictors of patient activation and attachment avoidance on the interest in e-health-related health applications, and significant influences of the predictor’s age and attachment avoidance on the use of e-health-related health applications were found. Higher patient activation is a well-known predictor for various interventions, especially if these require a certain self-management [40–42]. In the case of patients with an avoidant attachment, it was previously known that they tend to avoid personal GP contacts and have lower values in social support because they avoid close social relationships or acquiring help [43]. On the other hand, they seem to be particularly interested in e-health-related applications. This could mean that threatening health-related information is avoided if conveyed through another person, such as a GP. Telemedicine and app-based applications could be of relevance for this group of patients since these programs are run in a self-determined and autonomous manner [44]. The influence of avoidant attachment is also retained when using e-health-related health applications. This provides indications that this is actually a significant predictor, as a large number of other influences in the model were statistically excluded.

With regard to age, the statistically significant influence was only shown when using e-health-related health applications. This coincides with the literature, according to which older people have fewer opportunities to use these applications [45, 46]. Interestingly, general interest in e-health-related health applications showed no age influence. This indicates that older people are not fundamentally negative towards telemedicine either. In future studies, a distinction should therefore always be made between interest and use. A global explanation of an age effect in relation to e-health-related health applications would not be entirely correct. There are few gender effects in the present study. At least there is no statistical evidence of any influence of gender on the use and interest in telemedical applications. Gender influence is ambiguous in the literature. However, recent national studies also show no gender effect [16].

The exploratory calculations showed some final results. GPs generally seem to be a significant source of health information across all patient groups, which makes it clear that the influence of medical information is not lost, even if the Internet is also used. The general practitioner is well available because in Germany he or she is usually the gatekeeper in the medical care system and is consulted when symptoms occur. For women, pharmacies also seem to have an important advisory function. The pharmacist may be consulted in Germany when prescriptions are filled for medications or when non-prescription medications are purchased at the pharmacy. This is more of a proactive counseling process that the patient has to request. In general, only dosages and, if necessary, special intake conditions are otherwise communicated by the pharmacist. Moreover, older people are increasingly using medical journals for patients such as the ‘Apothekenrundschau’ (low-threshold information of a pharmacy journal aimed at patients) to seek medical advice. High-activated patients and patients with avoidant attachment use the smartphone more intensively to find out health-related information. The results fit in with the theoretical considerations according to which these people prefer less social contact, use less social support, and tend to avoid personal GP contacts.

Well-known socio-demographic influencing factors for the use of health-related internet and app-based applications could be confirmed. In addition, it has been shown that patient-related characteristics influence the level of interest and use of health-related e-health applications. In particular, patients with a high level of patient activation and avoidant attachment seem to particularly prefer mobile health applications. From an attachment perspective, it could be interesting to investigate in the future whether patients with, for example, avoidant attachment style prefer to acquire medical information using an online module and are more open to computer-mediated communication than patients with anxiety attachment, for whom the regular personal contact with a GP may be essential. In the future, we will also analyze specific health-risk behavior concerning their functions for different types of attachment.

However, in general, language barriers should be suppressed and improved in telemedical research in the future.

### Limitations

The sample was only recruited in one practice in Thuringia. It would, therefore, be interesting to transfer the survey to other federal states. Furthermore, it is a cross-sectional survey. Longitudinal surveys would also reveal information about the development of interests and changes in the usage behavior of e-health applications. Selection effects also resulted from the voluntary participation, although all patients were addressed on the reference dates. Persons with insufficient knowledge of German were excluded. Even if it was less than 1 % in the present study, it should be considered in the future how language barriers can be overcome and how telemedicine can also be implemented for patients with an intercultural background. Due to the rapid development in the field of telemedicine, the results cannot be carried over far into the future. Here it would be helpful to repeat such examinations in the course of the apps on prescription and an increase in video consultation hours.

In the area of the assessment of ties in adulthood, there are further problems to be discussed, which, for example, consist of a lack of convergence between the individual procedures, which operationalize the construct “attachment” dimensionally, categorically or according to the prototype approach and convert external or self-observation methods [47, 48]. The Weimar Telmed study primarily uses a dimensional self-assessment instrument ECR-RD12 with the primary subscales ‘attachment-related fear’ and ‘attachment-related avoidance’. The theoretical links with other study results are also made regarding other approaches in attachment research, which is not entirely unproblematic and an uncritical generalization should be avoided [49, 50].

The number of chronic illnesses per patient ranged from 0 to a maximum of 23. When asked about the current state of health, the patients reported a mean value of 64.2 on the visual analog scale with values from 0 to 100. A higher score indicates better self-rated health status. This also corresponds to studies in the field of primary care, although the number of type 2 diabetes patients should have been significantly higher [38, 51]. The explanation for this could be that the survey took place on specific fixed reference dates. The patients with type 2 diabetes took part to a large extent in Disease Management Programs (DMP) and were then specially invited by the staff to the DMP training courses, in which the doctor’s examinations were then planned in parallel to reduce travel for patients. Thus, by accident, they are less represented in the present study.

## Conclusion

Supportive e-health applications will become more and more important not only for patients with chronic illnesses but also in the context of health prevention. Provided that e-health offers are further established in the supply structure, they must also be more open to personalized approaches. For this purpose, questions about the needs of different types of patients must be taken into account. Since both the interest and the use of e-health applications differ depending on the type of attachment, these findings should be integrated into the health care system before health apps are randomly prescribed for all patients across the board. Depending on individual attachment needs, intervention programs could be carried out by computer or personally by the GP or with the involvement of a medical specialist.

## Data Availability

Study data are available at the Institute of Psychosocial Medicine, Psychotherapy and Psychooncology (Jena University Hospital). The datasets used and analysed during the current study are available from the corresponding author on reasonable request. After analysis of all data, they are deposited on zenodo https://zenodo.org.

## Abbreviations

GP: General practitioner
WHO: World Health Organisation
ECR: Experiences in Close Relationships
PAM: Patient Activation Measure
PHQ: Patient Health Questionnaire
DSM: Diagnostic and Statistical Manual
GLM: General Linear Model
E-Health: Electronic Health
VAS: Visual Analoge Scale
SPSS: Statistical Package for Social Sciences Statistics
